# Socioeconomic disparities in mortality from indoor air pollution: A multi-country study

**DOI:** 10.1371/journal.pone.0317581

**Published:** 2025-01-16

**Authors:** Muayad Albadrani

**Affiliations:** Department of Family and Community Medicine and Medical Education, College of Medicine, Taibah University, Al-Madinah Al-Munawara, Saudi Arabia; National Cheng Kung University, TAIWAN

## Abstract

**Background:**

Indoor air pollution is a major public health concern, contributing to approximately 2.9 million deaths and 81.1 million disability-adjusted life years lost annually. This issue disproportionately affects underprivileged communities that depend on solid fuels for cooking. As a result, these communities suffer from heightened exposure to indoor air pollutants, which increases the risk of morbidity, mortality, and worsening health disparities.

**Objective:**

This study investigates the association between socioeconomic status and mortality related to indoor air pollution across multiple countries.

**Methods:**

Data from the 2019 Demographic and Health Survey, WHO, and World Bank were utilized to examine the impact of socioeconomic status on indoor air pollution-related mortality. The primary outcome was mortality associated with solid fuel use, with income quintiles as the independent variable. Linear and logistic regression analyses were applied to assess these relationships.

**Results:**

Logistic regression analysis revealed a strong negative association where household income increases and indoor air pollution-related mortality significantly decreases. Specifically, Households in the highest income quartile showed a 22% reduction progressively in the odds of mortality risk compared to the lowest income quintile. Additionally, access to clean fuel correlated with a 0.59 times lower odds of mortality, highlighting the clean energy sources’ protecting effect.

**Conclusion:**

The findings highlight the critical need to prioritize clean fuel access, particularly in low-income communities, to reduce indoor air pollution mortality. Policies should focus on increasing clean energy accessibility and supporting vulnerable populations through targeted subsidies and poverty alleviation programs to reduce indoor air pollution exposure disparities.

## Introduction

Indoor air pollution is a critical public health issue, particularly in low- and middle-income countries, where socioeconomic disparities significantly influence exposure levels and associated health outcomes. The Environmental Protection Agency (EPA) defines environmental justice as ensuring fair treatment and meaningful involvement of all individuals, regardless of race, color, national origin, or income, in the development and enforcement of environmental policies [1, 2]. Despite this, vulnerable populations, especially in developing countries, face higher exposure to indoor air pollutants due to the widespread use of solid fuels for cooking. This exposure contributes to an estimated 3.2 million deaths annually, with the Global Burden of Disease (GBD) reporting 2.9 million deaths and 81.1 million disability-adjusted life years lost each year due to indoor air pollution [3]. Globally, approximately 3 billion people use solid fuels for cooking, which are an important source of indoor air pollution [3]. Indoor air can be negatively affected by outdoor pollution, particularly from human activities like traffic, which often introduces particulate matter (PM) and nitrogen dioxide (NO2) into indoor environments [4]. Once indoors, pollutants can change through chemical interactions with surfaces. For example, when nitrogen dioxide (NO2) reacts with various indoor materials, it can create nitrous acid (HONO), a pollutant associated with adverse health effects [5, 6]. Social and economic factors play a crucial role in determining the level of household exposure to indoor air pollution and illustrate that households in poor socioeconomic strata have a higher risk of morbidity and mortality due to several health conditions [7].

Several studies have examined the differences in harm from air pollution, influenced by factors such as race, ethnicity, socioeconomic position, education, and proximity to pollution sources [8–11]. The EPA found that the Black population faces a higher risk of particle pollution [12]. Various studies have explored differences in the effects of air pollution on early mortality. A recent study showed that those living in neighborhoods with a majority of Black residents within the Medicaid population had a higher risk of early death from particle pollution than those living in white neighborhoods [13]. Another study found that people of Hispanic, Asian, and Black descent had a higher risk of early mortality from particulate pollution than Whites, regardless of income. High-income African Americans still face chronic stress, potentially due to discrimination [14].

Therefore, there is still an ongoing debate regarding the specific reasons for these differences in mortality due to indoor air pollution. Another study found a higher risk for Black populations from harmful air pollutants, including traffic-related ones [15]. Years of residential discrimination and exclusion have led to the Black population living in areas with higher pollution exposure [16].

Household fuel type also influences indoor air pollution-related deaths. Because of the high cost of clean fuel, small, low-income households burn solid fuel, which exacerbates respiratory illnesses [3]. Therefore, a link between mortality from indoor air pollution and household socioeconomic status is expected owing to the higher use of unclean or solid fuels. Significant disparities exist in access to clean fuels and indoor air pollution within communities. Globally, approximately 60% of households have access to clean cooking fuel [17]. However, this varies according to the country’s wealth status or gross domestic product, as well as the socioeconomic status of the households within the country, especially in developing countries. Countries with a gross domestic product of less than $2,000 per year have poor access to clean energy, with only 10% of households having access to clean energy [17].

Indoor air pollution adversely affects health and leads to various health conditions, regardless of age [18]. This is an important cause of mortality, along with dietary risk factors, high blood pressure, and tobacco use [14]. Globally, indoor air pollution contributes to approximately 22–53% of all deaths from cardiovascular diseases, ischemic heart diseases, stroke, lung cancer, and chronic obstructive pulmonary disease (COPD) [19]. Immediate or long-term exposure to indoor air pollution can cause adverse health outcomes [20]. Immediate effects of indoor air pollution include eye, nose, and throat irritation, headache, rashes, sneezing, and fatigue. Long-term adverse effects include ischemic heart diseases, respiratory diseases such as COPD, lung cancer, stroke, and bronchial asthma [21]. Untreated, these health conditions can lead to increased mortality risk. The World Health Organization (WHO) reported that mortality due to indoor air pollution mainly occurs because of pneumonia, stroke, ischemic heart disease, chronic pulmonary disease, and lung cancer [21]. Different health outcomes have varying impacts on mortality based on age and sex. Determining the specific elements that affect indoor air pollution, like the density of outdoor traffic, allows for the design of focused interventions, which are often more effective in promoting health safety. However, relying on monitoring techniques can be complicated due to the diverse local conditions. For example, to assess how cooking appliances contribute to indoor pollution, it would be essential to evaluate window-opening habits in all households since proper ventilation is vital for reducing pollutants generated indoors [22].

While numerous studies have quantified the burden of indoor air pollution across countries, only a handful have specifically examined socioeconomic disparities in mortality resulting from health conditions related to indoor air pollution. Deprived communities globally bear higher pollution and outdoor air pollution, underscoring exposure disparities. Understanding the underlying drivers of these disparities is essential for designing effective interventions to reduce health inequities. Currently, there exists a dearth of evidence establishing a direct causal link between socioeconomic disparities and indoor air pollution-related mortality. The study aims to bridge this gap by investigating the relationship between socioeconomic factors and mortality due to indoor air pollution.

## Methods

The datasets utilized in this study are publicly available and have been anonymized by removing all individual-level identification variables. Consequently, the need for participant consent and ethical approval was deemed unnecessary. The study encompassed an analysis of populations across 217 countries, focusing on mortality attributed to indoor air pollution from solid fuel use. We employed data from the 2019 Demographic and Health Survey, WHO, and World Bank as covariates to assess their association with the health outcome. The World Bank data served as the source for the outcome variables. The primary dependent variables in regression models were the instances of mortality due to indoor air pollution across the 217 countries.

Mortality due to indoor air pollution from solid fuels quantifies the burden of mortality caused by indoor air pollution, specifically from cooking with solid fuels. Data on mortality due to indoor air pollution from solid fuels were obtained using three variables, mortality due to indoor air pollution, prevalence (proportion) of solid fuel usage for cooking in the country, and probability of death due to indoor air pollution caused by burning solid fuels obtained from the literature. Data on mortality owing to indoor air pollution were obtained from demographic and health surveys. Data on the prevalence of solid fuel cooking usage were obtained from the World Bank. All 217 countries were considered in this study. Only secondary data were used, which we obtained from various sources. No countries were excluded from the study except for those for which no data was available from either the World Bank or the Demographic and Health Survey Database.

**Fig 1:**
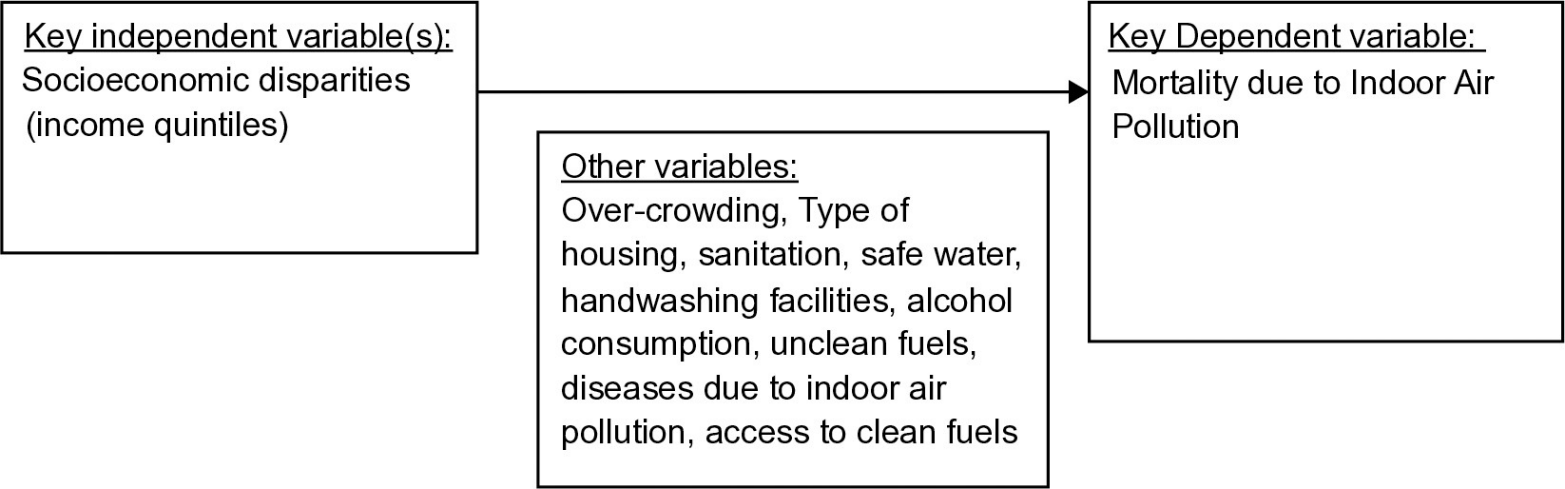
Analytic framework.

Fig 1 shows the analytical framework used to investigate the relationship between socioeconomic disparities and mortality due to indoor air pollution. The main independent variable was the income quintile (socioeconomic disparity). The possible covariates included household sanitation, clean cooking fuel, tobacco consumption, alcohol consumption, and overcrowding. We utilized a descriptive approach to delineate trends within the data. To investigate the effects of various independent variables on mortality outcomes due to indoor air pollution from solid fuel use, we employed linear regression (ordinary least squares; OLS) and logistic regression analyses. The linear regression analysis encompassed a series of tests to ensure the robustness and validity of research findings. These included goodness-of-fit hypothesis testing to evaluate the model’s fit, coefficient stability checks to confirm the reliability of the regression coefficients, and specification checks to verify the correct model specification. We assessed multicollinearity using the variance inflation factor and evaluated heteroscedasticity by plotting residuals against independent variables and predicted values. The hettest and White test in STATA, along with the Breusch–Pagan test, were utilized to further check for heteroscedasticity. Autocorrelation was examined through residual vs. time plots and the Durban–Watson test to maintain the independence assumption of the error terms under OLS regression. Newey–West standard errors were applied to correct for any identified heteroscedasticity and autocorrelation. A P-value of less than 0.05 was considered indicative of statistical significance in the analyses.

To avoid excluding countries from the analysis owing to the limited number of countries globally, an imputation method was used to handle the missing data. Imputation was performed by calculating the mean/median of the non-missing values in a column and then replacing the missing values within each column separately and independently. This was performed using the numerical data in this study. Imputation works well with small numerical datasets. Imputation was not performed by using the most frequent or zero-constant values; this method works well for categorical features by replacing the missing data with the most frequent values. We did not use this method because there were wide differences between the indicator values for countries for the different variables. Therefore, using the most frequent values may skew or bias study results. Considering the limitations of the data, other imputation methodologies, such as multivariate imputation using chained equations and imputation using Deep Learning, were not used in this study.

In this study, data management was meticulously conducted to ensure accuracy and reliability. Prior to analysis, data cleaning procedures were implemented to identify and correct any inconsistencies or missing values. The cleaned dataset was then subjected to a rigorous exploratory data analysis to understand the distribution of variables and identify potential outliers. To account for missing data, other measures to ensure data quality control were included. In this dataset, with a smaller sample size, outliers can bias the results of the study and affect its validity and interpretation. Outliers were identified using data visualization techniques, such as scatter plots, histograms, and box plots. Outliers were removed during post-test analysis. Trimmed estimators were used to obtain better and more robust statistics. Data were analyzed using STATA statistical software (Version 17).

For the statistical analysis, we employed logistic regression models to examine the relationship between socioeconomic status and mortality due to indoor air pollution. We ensured that the assumptions of logistic regression were met before interpreting the results. Statistical significance was determined using a two-tailed test with a significance level set at (p < 0.05). Results yielding a (p) -value below this threshold were considered statistically significant, indicating a less than 5% probability that the observed associations were due to chance.

**Fig 2:**
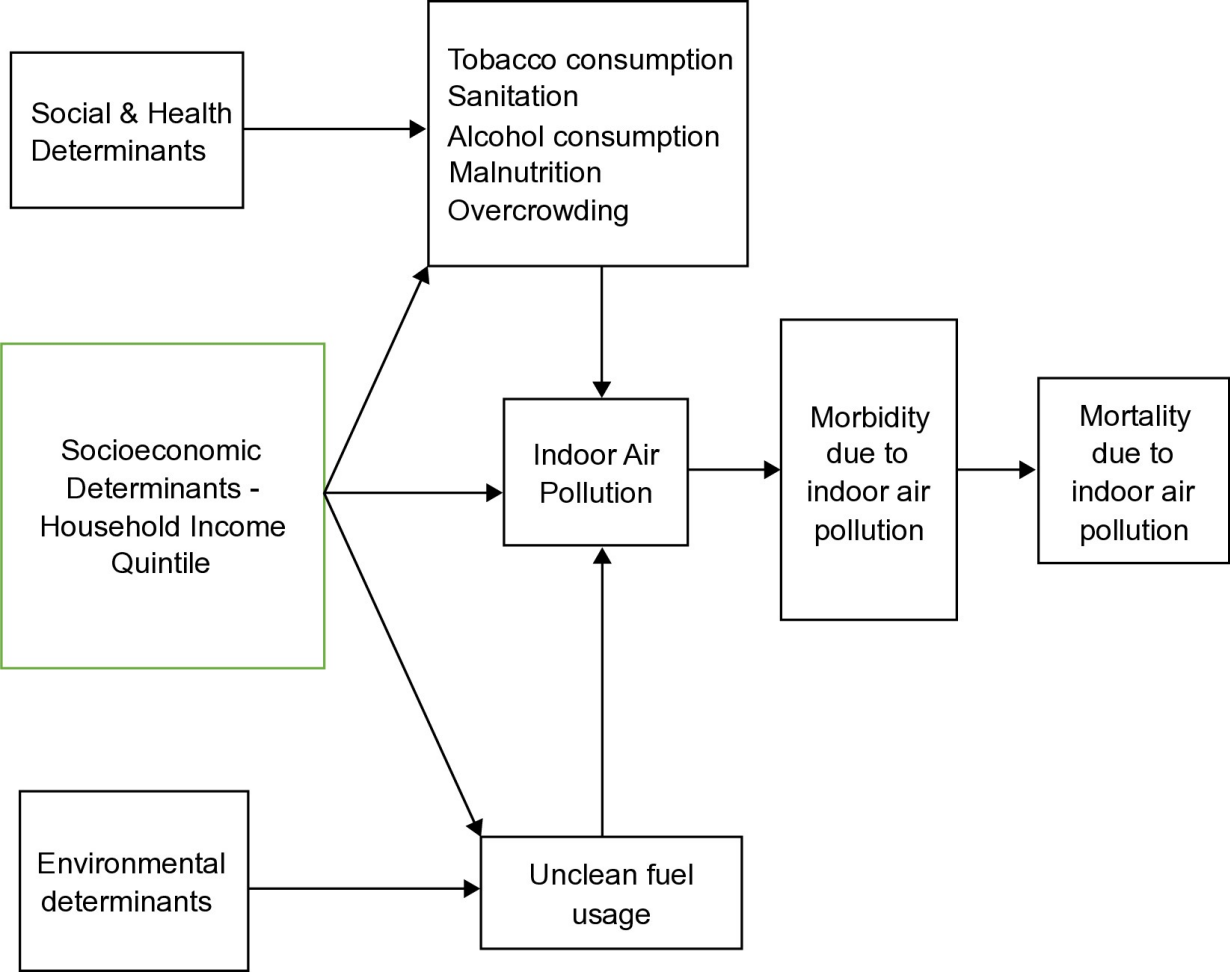
Diagrammatic framework of the determinants of mortality due to indoor air pollution.

The framework shown in Fig 2 illustrates the various determinants of mortality resulting from exposure to indoor air pollution, including social, health, socioeconomic, and environmental factors. This framework highlights the importance of considering various factors that influence indoor air quality and their impact on morbidity and mortality. Household income quintiles are associated with different social and health determinants of the household, such as tobacco and alcohol consumption, malnutrition, overcrowding, and sanitary conditions [23].

Malnutrition refers to deficiencies, excesses, or imbalances in an individual’s energy and/or nutrient intake [24, 25]. Here, malnutrition specifically refers to “underweight” (low weight for age), “stunting” (low height for age), and “wasting” (low weight for height). Overcrowding in households occurs when the number of occupants exceeds the dwelling space, as measured in terms of rooms, bedrooms, and flooring areas, leading to adverse physical and mental health outcomes. Safe sanitary conditions include access to clean drinking water services and safe disposal of wastewater and excreta, while poor sanitation increases the risk of diseases, malnutrition, and diarrhea.

Environmental determinants, such as unclean fuel usage in households, lead to increased indoor air pollution. Additionally, higher tobacco consumption, such as smoking, within households also increases indoor air pollution [3]. A rise in indoor air pollution prevalence results in increased morbidity, which may subsequently cause a surge in mortality due to indoor air pollution if not adequately treated or if complications from the illness worsen.

## Results

Table 1 illustrates the percentage of households facing indoor air pollution across income quintiles and regions, revealing significant disparities. Households in the lowest income quintile, particularly in rural areas, experience higher levels of pollution compared to those in urban regions.

**Table 1:** Percentage of households with indoor air pollution by income quintiles and regions.

Income Quintiles	Q1	26.0%
	Q2	22.6%
	Q3	19.6%
	Q4	15.8%
	Q5	16.0%
Region:	Rural	62.3%
	Urban	37.7%

*The percentages are for all countries combined

S1 Table provides insights into household income inequality across different countries. Notably, countries like Zambia have a significant proportion (61.3%) of households in the 5th (richest) income quintile, whereas countries such as Ukraine, Slovenia, Azerbaijan, the Czech Republic, and Moldova have a higher proportion (approximately 10%) of households in the 1st (poorest) income quintile.

S2 Table presents the global distribution of populations across urban and rural areas. It reveals that in countries like Kuwait, Monaco, and Singapore, nearly 100% of the population resides in urban areas, contrasting with countries like Papua New Guinea, where only 13% of the population lives in urban settings. Similarly, countries such as Rwanda and Samoa have predominantly rural populations (82%), while Uruguay has a predominantly urban population, with only 4% residing in rural areas.

Table 2 provides descriptive statistics for 217 countries, including key variables like indoor air pollution, tobacco use, access to clean fuels, poverty rates, and overcrowding. These statistics highlight the impact of socioeconomic factors on indoor air pollution exposure across regions.

**Table 2:** Descriptive statistics for the whole sample.

Variables	Definition	Mean (SD)
Indoor air pollution	Deaths due to indoor air pollution	18,179.80 (630.51)
Tobacco consumption	Tobacco consumption in the household (number of cigarettes per day per 100,000 population)	17.09 (3.34)
Clean fuels	Proportion of population having access to clean fuel for cooking (percentage of population)	27.93 (7.77)
Poverty	Poverty headcount ratio (percentage of population below national poverty lines)	33.17 (2.98)
Handwashing facilities	Population living in household with handwashing facility (percentage of population)	38.99 (5.67)
Overcrowding	Households with more than seven persons sleeping per room (percentage of population)	2.09 (0.75)
Alcohol consumption	Total alcohol consumption (in liters of pure alcohol)	4.35 (0.86)
Malnutrition	Prevalence of malnutrition (percentage of population)	13.33 (2.19)
Domestic sanitation	Proportion of population living in houses with dung floors (percentage of population)	2.12 (0.81)
Public Sanitation	Proportion of population using safely managed sanitation services (percentage of population)	35.33 (6.25)

S3 Table provides descriptive statistics of the variables by country. In some countries, such as India, household-attributable deaths due to air pollution from solid fuels are 811,262 per 100,000 population, whereas in other countries, such as Angola, they are 0 per 100,000 population. Access to clean fuel varied from 0% in Benin to 100% in Algeria. The poverty headcount varied from 76.4% in South Sudan to 0.6% in China. The prevalence of malnutrition varied from 43% in Madagascar to 3% in countries such as Sweden and New Zealand. In Uganda, 19.8% of the population lived in houses with dung floors, while 0.1% of the population in Afghanistan lived in houses with dung floors. In Nicaragua, 13.3% of households had more than seven persons sleeping per room, while only 0.1% of the households in Albania experienced overcrowding. The prevalence of tobacco use varied between 6.9% in Benin and 38.2% in Lebanon.

Table 3 presents the results of the linear regression analysis, demonstrating the significant relationship between socioeconomic status and mortality due to indoor air pollution from the use of solid fuels. Mortality was highest in the lowest income quintile, with a coefficient of 32.457 (p = 0.045), and progressively decreased across higher income quintiles, with the highest quintile showing a significant reduction in mortality (coefficient of 7.2637, p = 0.025). Additionally, access to clean fuel for cooking is significantly associated (p = 0.031) with reduced mortality rates, with a coefficient of -0.5631, serving as a protective factor.

**Table 3:** Linear regression analysis of the factors affecting mortality due to indoor air pollution due to the use of solid fuels by socioeconomic status.

Characteristics	Coefficient	Standard Error	P value
1^st^ quintile	32.4571	1.2443	0.045*
2^nd^ quintile	21.7892	1.3487	0.031*
3^rd^ quintile	13.2498	0.9873	0.024*
4^th^ quintile	11.5234	0.7646	0.038*
5^th^ quintile	7.2637	0.5682	0.025*
Access to clean fuel for cooking	-0.5631	0.2201	0.031*
Tobacco consumption in the household	11.2334	1.6279	0.534
Household with adequate handwashing facility	-2.5698	0.1283	0.349
Household with more than seven persons sleeping per room	27.2582	3.7419	0.672
Prevalence of malnutrition	1.4570	0.3276	0.256
Proportion of population living in houses with dung floors	7.3298	0.2789	0.214
Proportion of population using safely managed sanitation services	0.6782	0.4356	0.3475

*p<0.05 (statistically significant)

Table 4 summarizes the logistic regression analysis, which examines the odds of mortality due to indoor air pollution across different socioeconomic quintiles. Compared to the first quintile, households in the second quintile had significantly 0.76 times lower odds of mortality, p = 0.045. Households in the fourth quintile had a significant 0.32 times lower odds of mortality (p = 0.034), while those in the highest (fifth) quintile exhibited the lowest odds of mortality at 0.22 times (p = 0.023), indicating a substantial protective effect of higher socioeconomic status. Furthermore, households with access to clean fuel for cooking had significantly 0.59 times lower odds of mortality (p = 0.023).

**Table 4:** Logistic regression analysis of the factors affecting mortality due to indoor air pollution due to the use of solid fuels by socioeconomic status.

Characteristics	Odds Ratio	Standard Error	P value
2^nd^ quintile [Ref: 1^st^ quintile]	0.76	0.013	0.021*
3^rd^ quintile [Ref: 1^st^ quintile]	0.68	0.045	0.052
4^th^ quintile [Ref: 1^st^ quintile]	0.32	0.072	0.034*
5^th^ quintile [Ref: 1^st^ quintile]	0.22	0.235	0.014*
Households with access to clean fuel for cooking [Ref: households with no access to clean fuel]	0.59	0.023	0.023*
Tobacco consumption in the household [Ref: no tobacco consumption in household]	0.85	0.567	0.345
Household with adequate handwashing facility [Ref: household with no handwashing facility]	0.32	0.213	0.124
Household with more than seven persons sleeping per room [Ref: households with less than seven persons per room]	0.88	0.567	0.986
Living in houses with dung floors [Ref: not living in house with dung floors]	0.36	0.572	0.923

*p<0.05 (statistically significant)

## Discussion

This study highlights socioeconomic disparities in mortality due to indoor air pollution, revealing that individuals in the lowest income quintile are at significantly higher risk than wealthier groups. Lower-income households are more exposed to harmful pollutants from solid fuels used in cooking. This aligns with global evidence that shows disadvantaged populations face greater risks from fine particulate matter, leading to higher rates of premature death. For instance, studies conducted among 13.2 million Medicare enrollees in the United States (US) have demonstrated a similar trend, where lower socioeconomic groups face elevated risks of mortality due to air pollution [26]. In the US, lower-income populations and areas with high unemployment often face higher levels of particulate matter, increasing their health risks. This issue is also seen globally, where households use solid fuels like wood, crop waste, charcoal, coal, and dung. These fuels emit harmful pollutants such as carbon monoxide, formaldehyde, and nitrogen dioxide, which can lead to respiratory inflammation, weakened immune responses, and lower blood oxygen levels [27]. The health consequences are severe, contributing to the burden of diseases such as ischemic heart disease, stroke, lower respiratory infections, chronic obstructive pulmonary disease (COPD), and lung cancer. The disproportionate exposure and resulting health disparities among low-income households highlight the urgent need for targeted interventions to reduce indoor air pollution and its associated mortality [28].

A higher premature death risk was also observed in neighborhoods with higher unemployment rates and regions with greater transportation use, highlighting a poor socioeconomic status [28]. Another study found that increased indoor air pollution severity correlated with higher asthma-related mortality, particularly among poor and elderly populations [29]. There is also increased asthma-related mortality risk in neighborhoods with higher poverty rates, lower incomes, lower home values, and greater disparities [30–32] countries. The study did not analyze racial disparities; income and socioeconomic status were found to be vital determinants of mortality due to indoor air pollution, similar to the limited existing findings. This study, which used global data from all countries, also supports the evidence found in smaller studies conducted in different geographic locations.

Three main reasons for disparities are evident from the literature. First, there is increased exposure to indoor air pollution because of racism, class bias, and land costs. Households in disadvantaged communities are increasingly exposed to harmful outdoor and indoor air pollutants. Additionally, lower socioeconomic groups are already susceptible to health risks due to inadequate healthcare access and greater disparity. There are also differences in access to grocery stores, job opportunities, clean workplaces, and traffic exposure, disproportionally affecting populations facing higher inequality.

In the US, the unemployed and low-income groups live in areas with higher particle matter exposure. Globally, people of different incomes are exposed to different types of materials in their households, leading to varied health outcomes [33]. Primary sources of solid fuels include wood, crop waste, charcoal, coal, and dung. Indoor air pollution from solid fuels is caused by carbon monoxide, formaldehyde, pressed wood products, and nitrogen dioxide from burning solid fuels inside the household. The incomplete combustion of solid fuels, particulate matter, and other related pollutants causes inflammation in the airways and lungs, impairing immune response and reducing the oxygen-carrying capacity of the blood. Mortality due to indoor air pollution from solid fuel burning commonly occurs as a result of ischemic heart disease, stroke, lower respiratory infection, chronic obstructive pulmonary disease, and lung cancer.

The findings on the relationship between socioeconomic status and mortality due to indoor air pollution are consistent with research conducted in various parts of the world. In India, Balakrishnan et al. (2019) found that households in lower wealth quintiles had significantly higher exposure to particulate matter from solid fuel use, with concomitant increases in respiratory diseases [34]. Similarly, research in China by Yu et al. (2020) demonstrated that the use of solid fuels for cooking was associated with a 36% higher risk of cardiovascular mortality compared to clean fuel use, with the burden disproportionately affecting rural and less affluent populations [35].

In sub-Saharan Africa, a systematic review by Amegah and Jaakkola (2016) highlighted the pervasive use of biomass fuels in low-income households and its strong association with adverse health outcomes, particularly among women and children. The review emphasized the need for targeted interventions in these vulnerable populations [36]. Variations in indoor air quality can occur due to the architectural differences of buildings inhabited by different socioeconomic groups. Similarly, the behavior of occupants, which is shaped by social influences, can also contribute to this issue. Although research has not established a direct correlation between the act of opening windows and household income [37], individuals living in low socioeconomic status neighborhoods may be less likely to open their windows due to unfavorable perceptions of their environment. While studies that monitor exposure levels offer crucial evidence regarding inequalities, modeling studies can examine a broader array of scenarios and identify specific building characteristics and behaviors that might elevate indoor exposure levels [38].

The study showed that households with access to clean fuel for cooking have a decreased risk of death owing to indoor air pollution. The WHO reported that indoor air pollution causes approximately 3.2 million deaths per year, with a significant proportion being children under the age of 5 years [3]. Globally, evidence has shown that childhood mortality from respiratory infections is lower in countries with access to clean fuels [39]. Another study from China showed that the choice of cooking fuel is an important determinant of cardiopulmonary mortality, with the use of unclean fuels leading to increased mortality [40]. Another study conducted in Bangladesh showed that household air pollution affected children disproportionately, leading to an increase in deaths of children younger than 5 years in households that use unclean fuels [41]. Solid fuel use in households adversely affects pregnancy outcomes. A study conducted in Ghana showed that households using solid fuels had a higher risk of perinatal mortality, including several other adverse outcomes. Evidence suggests that childhood mortality rates from respiratory infections are lower in countries where clean fuels are more accessible [29]. Studies from China and Bangladesh further support this, showing that the choice of cooking fuel is a crucial determinant of cardiopulmonary mortality, with unclean fuels increasing the risk [30].

Various cooking methods can result in notable differences in the levels of particulate matter released indoors [42]. In this study, a single deterministic scenario was chosen to represent typical childhood occupancy patterns. Although this straightforward method simplifies the integration of human behavior into complex building simulations, it has certain drawbacks. On the other hand, a probabilistic approach acknowledges the unpredictable nature of occupant behavior, allowing for predictions of a range of possible outcomes while considering the variability in household occupancy and activities [43]. Dimitroulopoulou et al. utilized a probabilistic framework to assess personal exposure to indoor and outdoor air pollution for different population groups in both residential and non-residential settings, employing mass-balance micro-environmental models [44, 45]. Additionally, Milner et al. (2011) pointed out that incorporating probabilistic time-activity data into indoor air pollution models is an important focus for future research to enhance exposure estimates [46].

Future research should integrate qualitative data, like household SES, into quantitative models to assess exposure disparities across income groups. Advanced models such as EnergyPlus can evaluate exposure levels for different demographic segments, as individual behaviors influence time spent in various indoor environments. For instance, children from low-SES families may stay home more often due to negative perceptions of their neighborhood and a lack of activities [47]. The resulting exposure data can then be analyzed using models like the English Housing Survey, which provides information on income and smoking rates, to calculate exposure for a representative population [48].

The main strength of this study is the use of valid and reliable data from international agencies with robust data collection methods. However, a limitation of the study is its use of cross-sectional data, which can only establish associations and not causality. Future studies should focus on establishing causality rather than just associations, as highlighted in this study. Relevant multiyear data should be used in future studies to establish the socioeconomic disparities in mortality due to indoor air pollution. Future causal studies should help in developing specific interventions to reduce indoor air pollution and eliminate socioeconomic disparities.

## Conclusion

This study provides strong evidence that socioeconomic factors significantly reduce mortality caused by indoor air pollution. Logistic regression analysis showed that higher household income and access to clean cooking fuels are linked to lower odds of mortality. Specifically, individuals in the highest income quintile experienced a 22% reduction in mortality compared to those in the lowest quintile, while having access to clean fuel was associated with 0.59 times lower odds of mortality.

These findings highlight the urgent need to address socioeconomic disparities through targeted interventions, such as promoting clean energy solutions, implementing subsidies, and offering educational initiatives, especially in disadvantaged communities. By prioritizing these strategies, policymakers can effectively reduce the risks associated with indoor air pollution and improve health outcomes for vulnerable populations. Additionally, it is crucial to conduct experiments rigorously, incorporating appropriate controls, replication, and adequate sample sizes. This approach will validate the findings and strengthen the conclusions drawn, ultimately enhancing the reliability and accuracy of the research outcomes.

## Supporting information

S1 Table Household income inequality–composition by quintile. (DOCX)

S2 Table Urban–rural population by country. (DOCX)

S3 Table Descriptive statistics of independent variables by country. (DOCX)
