# Supplementary material for: Socioeconomic disparities in mortality from indoor air pollution: A multi-country study
Source: PLoS One. 2025 Jan 16;20(1):e0317581. doi: 10.1371/journal.pone.0317581 (PMC11737656; doi:10.1371/journal.pone.0317581)
Supplement: S1 Table — (DOCX) [file pone.0317581.s001.docx]

**S1 Table.** Household income inequality – composition by quintile

| S. No | Country | 1^st^ Quintile  (Lowest) | 2^nd^ Quintile  (2^nd^ Lowest) | 3^rd^ Quintile  (Middle) | 4^th^ Quintile  (2^nd^ Highest) | 5^th^ Quintile  (Highest) |
| --- | --- | --- | --- | --- | --- | --- |
| 1 | Afghanistan | 5.0 | 10.0 | 15.0 | 23.0 | 47.0 |
| 2 | Albania | 8.3 | 12.7 | 16.9 | 23.0 | 39.2 |
| 3 | Algeria | 9.4 | 13.7 | 17.5 | 22.3 | 37.2 |
| 4 | American Samoa |  |  |  |  |  |
| 5 | Andorra |  |  |  |  |  |
| 6 | Angola | 3.8 | 7.7 | 12.6 | 20.4 | 55.6 |
| 7 | Antigua and Barbuda |  |  |  |  |  |
| 8 | Argentina | 4.8 | 9.5 | 15.1 | 23.2 | 47.3 |
| 9 | Armenia | 10.2 | 14.3 | 17.8 | 22.2 | 35.6 |
| 10 | Aruba |  |  |  |  |  |
| 11 | Australia | 7.3 | 12.2 | 16.4 | 22.4 | 41.8 |
| 12 | Austria | 7.9 | 13.3 | 17.5 | 23.1 | 38.2 |
| 13 | Azerbaijan | 10.8 | 13.9 | 16.6 | 20.9 | 37.8 |
| 14 | The Bahamas |  |  |  |  |  |
| 15 | Bahrain |  |  |  |  |  |
| 16 | Bangladesh | 8.6 | 12.4 | 16.1 | 21.4 | 41.4 |
| 17 | Barbados |  |  |  |  |  |
| 18 | Belarus | 10.3 | 14.5 | 18.0 | 22.5 | 34.8 |
| 19 | Belgium | 9.0 | 14.2 | 17.9 | 22.3 | 36.5 |
| 20 | Belize | 3.2 | 7.7 | 12.0 | 19.4 | 57.7 |
| 21 | Benin | 7.0 | 11.1 | 15.0 | 21.4 | 45.4 |
| 22 | Bermuda |  |  |  |  |  |
| 23 | Bhutan | 6.7 | 10.8 | 15.5 | 22.7 | 44.4 |
| 24 | Bolivia | 4.7 | 9.8 | 14.5 | 22.0 | 49.0 |
| 25 | Bosnia and Herzegovina | 7.5 | 12.3 | 16.7 | 22.9 | 40.7 |
| 26 | Botswana | 3.9 | 7.0 | 11.1 | 19.5 | 58.5 |
| 27 | Brazil | 4.5 | 8.7 | 12.9 | 19.3 | 54.7 |
| 28 | British Virgin Islands |  |  |  |  |  |
| 29 | Brunei Darussalam |  |  |  |  |  |
| 30 | Bulgaria | 5.7 | 11.0 | 15.2 | 21.4 | 46.6 |
| 31 | Burkina Faso | 5.5 | 8.6 | 12.2 | 19.5 | 54.3 |
| 32 | Burundi | 6.9 | 11.0 | 15.0 | 20.8 | 46.3 |
| 33 | Cabo Verde | 5.7 | 9.7 | 14.3 | 21.6 | 48.7 |
| 34 | Cambodia |  |  |  |  |  |
| 35 | Cameroon | 4.5 | 8.5 | 13.7 | 21.6 | 51.7 |
| 36 | Canada | 7.1 | 12.4 | 17.0 | 22.9 | 40.6 |
| 37 | Cayman Islands |  |  |  |  |  |
| 38 | Central African Republic |  | 7.0 | 11.1 | 17.7 | 60.9 |
| 39 | Chad |  | 11.1 | 15.2 | 21.4 | 45.2 |
| 40 | Channel Islands |  |  |  |  |  |
| 41 | Chile | 5.5 | 9.5 | 13.5 | 19.9 | 51.6 |
| 42 | China | 6.7 | 10.7 | 15.2 | 22.0 | 45.3 |
| 43 | Colombia | 2.8 | 7.4 | 11.9 | 19.6 | 58.3 |
| 44 | Comoros | 4.5 | 9.1 | 13.9 | 22.2 | 50.4 |
| 45 | Dem. Rep. Congo | 5.5 | 10.0 | 14.5 | 21.6 | 48.4 |
| 46 | Congo, Rep. | 4.2 | 8.2 | 13.2 | 20.7 | 53.7 |
| 47 | Costa Rica | 4.0 | 8.1 | 12.6 | 20.9 | 54.4 |
| 48 | Cote d'Ivoire | 7.0 | 11.0 | 15.4 | 21.8 | 44.7 |
| 49 | Croatia | 8.0 | 13.7 | 18.1 | 23.3 | 37.0 |
| 50 | Cuba |  |  |  |  |  |
| 51 | Curacao |  |  |  |  |  |
| 52 | Cyprus | 8.5 | 12.8 | 16.7 | 21.8 | 40.1 |
| 53 | Czech Republic | 10.1 | 14.5 | 17.6 | 22.1 | 35.7 |
| 54 | Denmark | 9.5 | 14.0 | 17.3 | 21.9 | 37.3 |
| 55 | Djibouti | 5.4 | 10.4 | 15.1 | 21.5 | 47.6 |
| 56 | Dominica |  |  |  |  |  |
| 57 | Dominican Republic | 6.2 | 10.7 | 14.9 | 21.7 | 46.5 |
| 58 | Ecuador | 4.1 | 8.9 | 13.6 | 21.2 | 52.3 |
| 59 | Egypt, Arab Rep. | 9.0 | 12.8 | 16.2 | 21.0 | 41.0 |
| 60 | El Salvador | 6.3 | 10.8 | 15.4 | 21.9 | 45.6 |
| 61 | Equatorial Guinea |  |  |  |  |  |
| 62 | Eritrea |  |  |  |  |  |
| 63 | Estonia | 8.1 | 12.9 | 16.9 | 23.2 | 38.9 |
| 64 | Eswatini | 3.7 | 6.8 | 10.7 | 18.6 | 60.2 |
| 65 | Ethiopia | 7.3 | 12.1 | 16.3 | 21.2 | 43.0 |
| 66 | Faroe Islands |  |  |  |  |  |
| 67 | Fiji | 8.4 | 12.9 | 16.8 | 22.5 | 39.4 |
| 68 | Finland | 9.2 | 13.9 | 17.4 | 22.3 | 37.1 |
| 69 | France | 8.0 | 12.9 | 16.7 | 21.6 | 40.8 |
| 70 | French Polynesia |  |  |  |  |  |
| 71 | Gabon | 6.0 | 10.8 | 15.7 | 23.0 | 44.4 |
| 72 | Gambia, The | 7.4 | 11.6 | 15.7 | 21.8 | 43.6 |
| 73 | Georgia | 7.0 | 12.0 | 16.7 | 22.9 | 41.5 |
| 74 | Germany | 7.9 | 12.9 | 17.0 | 22.3 | 39.9 |
| 75 | Ghana | 4.7 | 9.6 | 14.8 | 22.3 | 48.6 |
| 76 | Gibraltar |  |  |  |  |  |
| 77 | Greece | 7.0 | 12.6 | 17.2 | 23.1 | 40.1 |
| 78 | Greenland |  |  |  |  |  |
| 79 | Grenada |  |  |  |  |  |
| 80 | Guam |  |  |  |  |  |
| 81 | Guatemala | 4.5 | 8.6 | 13.2 | 20.1 | 53.6 |
| 82 | Guinea | 8.5 | 13.1 | 17.3 | 22.9 | 38.2 |
| 83 | Guinea-Bissau | 7.8 | 11.6 | 15.7 | 21.8 | 43.0 |
| 84 | Guyana | 4.2 | 9.8 | 14.5 | 21.3 | 50.2 |
| 85 | Haiti | 5.5 | 10.3 | 15.2 | 21.9 | 47.1 |
| 86 | Honduras | 3.6 | 8.0 | 13.7 | 22.5 | 52.2 |
| 87 | Hong Kong SAR, China |  |  |  |  |  |
| 88 | Hungary | 8.0 | 13.4 | 17.7 | 23.1 | 37.8 |
| 89 | Iceland | 9.7 | 14.2 | 17.9 | 22.3 | 35.9 |
| 90 | India | 8.1 | 11.8 | 15.2 | 20.1 | 44.7 |
| 91 | Indonesia | 7.0 | 10.9 | 15.1 | 21.5 | 45.4 |
| 92 | Iran, Islamic Rep. | 6.0 | 10.3 | 14.7 | 21.6 | 47.5 |
| 93 | Iraq | 8.8 | 13.1 | 17.1 | 22.5 | 38.5 |
| 94 | Ireland | 8.7 | 13.1 | 16.6 | 21.9 | 39.7 |
| 95 | Isle of Man |  |  |  |  |  |
| 96 | Israel | 5.2 | 10.9 | 16.5 | 23.6 | 43.8 |
| 97 | Italy | 6.1 | 12.2 | 17.0 | 23.2 | 41.5 |
| 98 | Jamaica | 5.3 | 9.2 | 13.2 | 20.6 | 51.6 |
| 99 | Japan | 7.7 | 12.8 | 16.6 | 21.7 | 41.1 |
| 100 | Jordan | 8.2 | 12.1 | 15.8 | 21.5 | 42.4 |
| 101 | Kazakhstan | 9.8 | 13.5 | 16.8 | 21.9 | 37.9 |
| 102 | Kenya | 6.2 | 10.3 | 14.6 | 21.5 | 47.5 |
| 103 | Kiribati | 9.5 | 13.5 | 17.2 | 22.5 | 37.4 |
| 104 | Korea, Dem. People's Rep. |  |  |  |  |  |
| 105 | Korea, Rep. | 7.5 | 13.0 | 17.4 | 23.0 | 39.1 |
| 106 | Kosovo | 9.2 | 13.6 | 17.0 | 21.6 | 38.6 |
| 107 | Kuwait |  |  |  |  |  |
| 108 | Kyrgyz Republic | 9.4 | 13.1 | 16.9 | 22.1 | 38.5 |
| 109 | Lao PDR | 7.0 | 10.8 | 14.7 | 21.0 | 46.4 |
| 110 | Latvia | 7.1 | 12.2 | 16.4 | 22.3 | 42.0 |
| 111 | Lebanon | 7.9 | 12.7 | 17.0 | 22.5 | 40.0 |
| 112 | Lesotho | 4.6 | 8.9 | 14.2 | 22.6 | 49.8 |
| 113 | Liberia | 7.2 | 11.6 | 16.0 | 22.3 | 42.8 |
| 114 | Libya |  |  |  |  |  |
| 115 | Liechtenstein |  |  |  |  |  |
| 116 | Lithuania | 7.0 | 12.0 | 16.1 | 22.1 | 42.8 |
| 117 | Luxembourg | 7.2 | 11.9 | 16.5 | 23.0 | 41.4 |
| 118 | Macao SAR, China |  |  |  |  |  |
| 119 | Madagascar | 5.7 | 10.0 | 14.1 | 20.7 | 49.4 |
| 120 | Malawi | 6.9 | 11.0 | 14.9 | 21.0 | 46.2 |
| 121 | Malaysia | 5.8 | 10.1 | 14.8 | 22.0 | 47.3 |
| 122 | Maldives | 8.9 | 13.2 | 17.0 | 22.4 | 38.5 |
| 123 | Mali | 7.5 | 11.2 | 15.3 | 22.1 | 43.9 |
| 124 | Malta | 8.1 | 13.1 | 16.9 | 22.5 | 39.4 |
| 125 | Marshall Islands | 7.2 | 11.7 | 15.8 | 22.0 | 43.3 |
| 126 | Mauritania | 7.5 | 12.4 | 16.9 | 23.0 | 40.2 |
| 127 | Mauritius | 7.2 | 11.6 | 15.5 | 21.1 | 44.6 |
| 128 | Mexico | 5.0 | 9.3 | 13.8 | 20.7 | 51.2 |
| 129 | Micronesia, Fed. Sts. | 5.5 | 10.7 | 15.3 | 22.5 | 46.0 |
| 130 | Moldova | 10.0 | 14.1 | 17.5 | 22.3 | 36.2 |
| 131 | Monaco |  |  |  |  |  |
| 132 | Mongolia | 7.9 | 12.3 | 16.5 | 22.5 | 40.9 |
| 133 | Montenegro | 5.3 | 11.5 | 17.1 | 23.8 | 42.3 |
| 134 | Morocco | 6.7 | 10.7 | 14.7 | 20.9 | 47.0 |
| 135 | Mozambique | 4.2 | 7.6 | 11.2 | 17.4 | 59.5 |
| 136 | Myanmar | 8.9 | 13.0 | 16.6 | 21.6 | 39.9 |
| 137 | Namibia | 2.8 | 5.8 | 9.8 | 17.9 | 63.7 |
| 138 | Nauru | 7.5 | 11.9 | 15.9 | 22.0 | 42.7 |
| 139 | Nepal | 8.3 | 12.1 | 16.2 | 21.8 | 41.5 |
| 140 | Netherlands | 8.6 | 13.7 | 17.4 | 22.2 | 38.0 |
| 141 | New Caledonia |  |  |  |  |  |
| 142 | New Zealand |  |  |  |  |  |
| 143 | Nicaragua | 5.1 | 9.2 | 13.7 | 20.0 | 52.1 |
| 144 | Niger | 7.6 | 11.4 | 15.0 | 20.4 | 45.7 |
| 145 | Nigeria | 7.1 | 11.6 | 16.2 | 22.7 | 42.4 |
| 146 | North Macedonia | 6.1 | 12.4 | 18.1 | 24.6 | 38.8 |
| 147 | Northern Mariana Islands |  |  |  |  |  |
| 148 | Norway | 8.8 | 14.1 | 17.7 | 22.6 | 36.7 |
| 149 | Oman |  |  |  |  |  |
| 150 | Pakistan | 9.6 | 13.1 | 16.4 | 21.2 | 39.6 |
| 151 | Palau |  |  |  |  |  |
| 152 | Panama | 3.6 | 8.1 | 13.2 | 20.7 | 54.4 |
| 153 | Papua New Guinea | 5.1 | 10.0 | 15.2 | 22.4 | 47.3 |
| 154 | Paraguay | 5.3 | 9.6 | 14.4 | 21.5 | 49.3 |
| 155 | Peru | 4.8 | 9.6 | 14.6 | 21.9 | 49.1 |
| 156 | Philippines | 6.2 | 9.9 | 14.0 | 20.7 | 49.2 |
| 157 | Poland | 8.2 | 13.3 | 17.4 | 22.5 | 38.6 |
| 158 | Portugal | 7.6 | 12.8 | 16.7 | 22.0 | 40.9 |
| 159 | Puerto Rico |  |  |  |  |  |
| 160 | Qatar |  |  |  |  |  |
| 161 | Romania | 5.7 | 12.1 | 17.7 | 24.2 | 40.4 |
| 162 | Russian Federation | 7.5 | 11.6 | 15.5 | 21.4 | 44.0 |
| 163 | Rwanda | 6.0 | 9.8 | 13.6 | 19.8 | 50.8 |
| 164 | Samoa | 6.8 | 11.1 | 14.9 | 20.8 | 46.4 |
| 165 | San Marino |  |  |  |  |  |
| 166 | Sao Tome and Principe | 6.4 | 10.4 | 14.6 | 20.7 | 47.9 |
| 167 | Saudi Arabia |  |  |  |  |  |
| 168 | Senegal | 7.1 | 10.9 | 15.0 | 21.3 | 45.8 |
| 169 | Serbia | 6.4 | 12.5 | 17.1 | 22.9 | 41.2 |
| 170 | Seychelles | 7.0 | 12.6 | 17.6 | 23.6 | 39.1 |
| 171 | Sierra Leone | 7.9 | 11.7 | 15.3 | 21.0 | 44.2 |
| 172 | Singapore |  |  |  |  |  |
| 173 | Sint Maarten (Dutch part) |  |  |  |  |  |
| 174 | Slovak Republic | 9.5 | 15.4 | 19.0 | 23.2 | 32.8 |
| 175 | Slovenia | 10.1 | 14.7 | 18.2 | 22.4 | 34.6 |
| 176 | Solomon Islands | 7.0 | 11.4 | 15.5 | 21.5 | 44.6 |
| 177 | Somalia |  |  |  |  |  |
| 178 | South Africa | 2.4 | 4.8 | 8.2 | 16.5 | 68.2 |
| 179 | South Sudan | 3.9 | 8.6 | 14.2 | 22.8 | 50.6 |
| 180 | Spain | 6.2 | 12.3 | 17.3 | 23.6 | 40.6 |
| 181 | Sri Lanka | 7.1 | 10.8 | 14.6 | 20.3 | 47.2 |
| 182 | St. Kitts and Nevis |  |  |  |  |  |
| 183 | St. Lucia | 3.1 | 7.9 | 13.0 | 20.6 | 55.4 |
| 184 | St. Martin (French part) |  |  |  |  |  |
| 185 | St. Vincent and the Grenadines |  |  |  |  |  |
| 186 | Sudan | 7.8 | 12.1 | 16.1 | 21.6 | 42.4 |
| 187 | Suriname |  | 6.2 | 12.2 | 20.0 | 60.5 |
| 188 | Sweden | 8.1 | 13.8 | 17.6 | 23.0 | 37.5 |
| 189 | Switzerland | 7.5 | 12.4 | 16.8 | 22.5 | 40.8 |
| 190 | Syrian Arab Republic | 7.2 | 11.1 | 15.0 | 21.3 | 45.3 |
| 191 | Tajikistan | 7.4 | 12.0 | 16.4 | 22.4 | 41.7 |
| 192 | Tanzania | 6.9 | 10.5 | 14.2 | 20.4 | 48.1 |
| 193 | Thailand | 7.5 | 11.5 | 15.8 | 22.4 | 42.7 |
| 194 | Timor-Leste | 9.4 | 13.4 | 16.9 | 21.8 | 38.4 |
| 195 | Togo | 5.8 | 9.9 | 14.2 | 21.2 | 48.9 |
| 196 | Tonga | 6.8 | 11.4 | 15.2 | 21.2 | 45.4 |
| 197 | Trinidad and Tobago | 5.5 | 10.3 | 15.5 | 22.7 | 45.9 |
| 198 | Tunisia | 7.8 | 12.3 | 16.5 | 22.5 | 40.9 |
| 199 | Turkey | 5.4 | 10.1 | 14.6 | 21.8 | 48.0 |
| 200 | Turkmenistan | 6.1 | 10.3 | 14.5 | 21.6 | 47.5 |
| 201 | Turks and Caicos Islands |  |  |  |  |  |
| 202 | Tuvalu | 6.6 | 10.8 | 14.9 | 21.3 | 46.4 |
| 203 | Uganda | 6.1 | 10.0 | 14.1 | 20.3 | 49.5 |
| 204 | Ukraine | 10.0 | 14.3 | 17.6 | 22.1 | 35.9 |
| 205 | United Arab Emirates | 9.2 | 13.8 | 18.3 | 23.9 | 34.8 |
| 206 | United Kingdom | 6.8 | 11.8 | 16.5 | 22.8 | 42.1 |
| 207 | United States | 5.1 | 10.2 | 15.2 | 22.5 | 47.0 |
| 208 | Uruguay | 5.7 | 12.0 | 15.3 | 22.5 | 46.2 |
| 209 | Uzbekistan | 7.4 | 12.4 | 15.8 | 21.4 | 43.4 |
| 210 | Vanuatu | 7.5 | 12.4 | 17.2 | 23.0 | 39.9 |
| 211 | Venezuela, RB | 4.2 | 9.6 | 14.6 | 22.1 | 49.5 |
| 212 | Vietnam | 6.7 | 11.9 | 16.3 | 22.3 | 42.9 |
| 213 | Virgin Islands (U.S.) |  |  |  |  |  |
| 214 | West Bank and Gaza | 7.3 | 11.9 | 16.6 | 23.2 | 41.1 |
| 215 | Yemen, Rep. | 7.3 | 11.5 | 15.3 | 21.2 | 44.7 |
| 216 | Zambia | 2.9 | 6.0 | 10.6 | 19.3 | 61.3 |
| 217 | Zimbabwe | 6.0 | 9.1 | 13.2 | 20.6 | 51.1 |
