# Supplementary material for: Socioeconomic disparities in mortality from indoor air pollution: A multi-country study
Source: PLoS One. 2025 Jan 16;20(1):e0317581. doi: 10.1371/journal.pone.0317581 (PMC11737656; doi:10.1371/journal.pone.0317581)
Supplement: S2 Table — (DOCX) [file pone.0317581.s002.docx]

**S2 Table.** Urban–rural population by country

| S. No | Country | Urban Population | Rural Population |
| --- | --- | --- | --- |
| 1 | Afghanistan | 26 | 74 |
| 2 | Albania | 63 | 37 |
| 3 | Algeria | 74 | 26 |
| 4 | American Samoa | 87 | 13 |
| 5 | Andorra | 88 | 12 |
| 6 | Angola | 67 | 33 |
| 7 | Antigua and Barbuda | 24 | 76 |
| 8 | Argentina | 92 | 8 |
| 9 | Armenia | 63 | 37 |
| 10 | Aruba | 44 | 56 |
| 11 | Australia | 86 | 14 |
| 12 | Austria | 59 | 41 |
| 13 | Azerbaijan | 57 | 43 |
| 14 | The Bahamas | 83 | 17 |
| 15 | Bahrain | 90 | 10 |
| 16 | Bangladesh | 39 | 61 |
| 17 | Barbados | 31 | 69 |
| 18 | Belarus | 80 | 20 |
| 19 | Belgium | 98 | 2 |
| 20 | Belize | 46 | 54 |
| 21 | Benin | 49 | 51 |
| 22 | Bermuda | 100 | 0 |
| 23 | Bhutan | 43 | 57 |
| 24 | Bolivia | 70 | 30 |
| 25 | Bosnia and Herzegovina | 49 | 51 |
| 26 | Botswana | 72 | 28 |
| 27 | Brazil | 87 | 13 |
| 28 | British Virgin Islands | 49 | 51 |
| 29 | Brunei Darussalam | 79 | 21 |
| 30 | Bulgaria | 76 | 24 |
| 31 | Burkina Faso | 31 | 69 |
| 32 | Burundi | 14 | 86 |
| 33 | Cabo Verde | 67 | 33 |
| 34 | Cambodia | 25 | 75 |
| 35 | Cameroon | 58 | 42 |
| 36 | Canada | 82 | 18 |
| 37 | Cayman Islands | 100 | 0 |
| 38 | Central African Republic | 43 | 57 |
| 39 | Chad | 24 | 76 |
| 40 | Channel Islands | 31 | 69 |
| 41 | Chile | 88 | 12 |
| 42 | China | 63 | 37 |
| 43 | Colombia | 82 | 18 |
| 44 | Comoros | 30 | 70 |
| 45 | Dem. Rep. Congo | 46 | 54 |
| 46 | Congo, Rep. | 68 | 32 |
| 47 | Costa Rica | 81 | 19 |
| 48 | Cote d'Ivoire | 52 | 48 |
| 49 | Croatia | 58 | 42 |
| 50 | Cuba | 77 | 23 |
| 51 | Curacao | 89 | 11 |
| 52 | Cyprus | 67 | 33 |
| 53 | Czech Republic | 74 | 26 |
| 54 | Denmark | 88 | 12 |
| 55 | Djibouti | 78 | 22 |
| 56 | Dominica | 71 | 29 |
| 57 | Dominican Republic | 83 | 17 |
| 58 | Ecuador | 64 | 36 |
| 59 | Egypt, Arab Rep. | 43 | 57 |
| 60 | El Salvador | 74 | 26 |
| 61 | Equatorial Guinea | 74 | 26 |
| 62 | Eritrea | 36 | 64 |
| 63 | Estonia | 69 | 31 |
| 64 | Eswatini | 24 | 76 |
| 65 | Ethiopia | 22 | 78 |
| 66 | Faroe Islands | 43 | 57 |
| 67 | Fiji | 58 | 42 |
| 68 | Finland | 86 | 14 |
| 69 | France | 81 | 19 |
| 70 | French Polynesia | 62 | 38 |
| 71 | Gabon | 90 | 10 |
| 72 | Gambia, The | 63 | 37 |
| 73 | Georgia | 60 | 40 |
| 74 | Germany | 78 | 22 |
| 75 | Ghana | 58 | 42 |
| 76 | Gibraltar | 100 | 0 |
| 77 | Greece | 80 | 20 |
| 78 | Greenland | 88 | 12 |
| 79 | Grenada | 37 | 63 |
| 80 | Guam | 95 | 5 |
| 81 | Guatemala | 52 | 48 |
| 82 | Guinea | 37 | 63 |
| 83 | Guinea-Bissau | 45 | 55 |
| 84 | Guyana | 27 | 73 |
| 85 | Haiti | 58 | 42 |
| 86 | Honduras | 59 | 41 |
| 87 | Hong Kong SAR, China | 100 | 0 |
| 88 | Hungary | 72 | 28 |
| 89 | Iceland | 94 | 6 |
| 90 | India | 35 | 65 |
| 91 | Indonesia | 57 | 43 |
| 92 | Iran, Islamic Rep. | 76 | 24 |
| 93 | Iraq | 71 | 29 |
| 94 | Ireland | 64 | 36 |
| 95 | Isle of Man | 53 | 47 |
| 96 | Israel | 93 | 7 |
| 97 | Italy | 71 | 29 |
| 98 | Jamaica | 57 | 43 |
| 99 | Japan | 92 | 8 |
| 100 | Jordan | 92 | 8 |
| 101 | Kazakhstan | 58 | 42 |
| 102 | Kenya | 28 | 72 |
| 103 | Kiribati | 56 | 44 |
| 104 | Korea, Dem. People's Rep. | 63 | 37 |
| 105 | Korea, Rep. | 81 | 19 |
| 106 | Kosovo |  |  |
| 107 | Kuwait | 100 | 0 |
| 108 | Kyrgyz Republic | 37 | 63 |
| 109 | Lao PDR | 37 | 63 |
| 110 | Latvia | 68 | 32 |
| 111 | Lebanon | 89 | 11 |
| 112 | Lesotho | 29 | 71 |
| 113 | Liberia | 53 | 47 |
| 114 | Libya | 81 | 19 |
| 115 | Liechtenstein | 14 | 86 |
| 116 | Lithuania | 68 | 32 |
| 117 | Luxembourg | 92 | 8 |
| 118 | Macao SAR, China | 100 | 0 |
| 119 | Madagascar | 39 | 61 |
| 120 | Malawi | 18 | 82 |
| 121 | Malaysia | 78 | 22 |
| 122 | Maldives | 41 | 59 |
| 123 | Mali | 45 | 55 |
| 124 | Malta | 95 | 5 |
| 125 | Marshall Islands | 78 | 22 |
| 126 | Mauritania | 56 | 44 |
| 127 | Mauritius | 41 | 59 |
| 128 | Mexico | 81 | 19 |
| 129 | Micronesia, Fed. Sts. | 23 | 77 |
| 130 | Moldova | 43 | 57 |
| 131 | Monaco | 100 | 0 |
| 132 | Mongolia | 69 | 31 |
| 133 | Montenegro | 68 | 32 |
| 134 | Morocco | 64 | 36 |
| 135 | Mozambique | 38 | 62 |
| 136 | Myanmar | 31 | 69 |
| 137 | Namibia | 53 | 47 |
| 138 | Nauru | 100 | 0 |
| 139 | Nepal | 21 | 79 |
| 140 | Netherlands | 93 | 7 |
| 141 | New Caledonia | 72 | 28 |
| 142 | New Zealand | 87 | 13 |
| 143 | Nicaragua | 59 | 41 |
| 144 | Niger | 17 | 83 |
| 145 | Nigeria | 53 | 47 |
| 146 | North Macedonia | 59 | 41 |
| 147 | Northern Mariana Islands | 92 | 8 |
| 148 | Norway | 83 | 17 |
| 149 | Oman | 87 | 13 |
| 150 | Pakistan | 37 | 63 |
| 151 | Palau | 81 | 19 |
| 152 | Panama | 69 | 31 |
| 153 | Papua New Guinea | 13 | 87 |
| 154 | Paraguay | 62 | 38 |
| 155 | Peru | 79 | 21 |
| 156 | Philippines | 48 | 52 |
| 157 | Poland | 60 | 40 |
| 158 | Portugal | 67 | 33 |
| 159 | Puerto Rico | 94 | 6 |
| 160 | Qatar | 99 | 1 |
| 161 | Romania | 54 | 46 |
| 162 | Russian Federation | 75 | 25 |
| 163 | Rwanda | 18 | 82 |
| 164 | Samoa | 18 | 82 |
| 165 | San Marino | 98 | 2 |
| 166 | Sao Tome and Principe | 75 | 25 |
| 167 | Saudi Arabia | 85 | 15 |
| 168 | Senegal | 49 | 51 |
| 169 | Serbia | 57 | 43 |
| 170 | Seychelles | 58 | 42 |
| 171 | Sierra Leone | 43 | 57 |
| 172 | Singapore | 100 | 0 |
| 173 | Sint Maarten (Dutch part) | 100 | 0 |
| 174 | Slovak Republic | 54 | 46 |
| 175 | Slovenia | 55 | 45 |
| 176 | Solomon Islands | 25 | 75 |
| 177 | Somalia | 47 | 53 |
| 178 | South Africa | 68 | 32 |
| 179 | South Sudan | 21 | 79 |
| 180 | Spain | 81 | 19 |
| 181 | Sri Lanka | 19 | 81 |
| 182 | St. Kitts and Nevis | 31 | 69 |
| 183 | St. Lucia | 19 | 81 |
| 184 | St. Martin (French part) |  |  |
| 185 | St. Vincent and the Grenadines | 53 | 47 |
| 186 | Sudan | 36 | 64 |
| 187 | Suriname | 66 | 34 |
| 188 | Sweden | 88 | 12 |
| 189 | Switzerland | 74 | 26 |
| 190 | Syrian Arab Republic | 56 | 44 |
| 191 | Tajikistan | 28 | 72 |
| 192 | Tanzania | 36 | 64 |
| 193 | Thailand | 52 | 48 |
| 194 | Timor-Leste | 32 | 68 |
| 195 | Togo | 43 | 57 |
| 196 | Tonga | 23 | 77 |
| 197 | Trinidad and Tobago | 53 | 47 |
| 198 | Tunisia | 70 | 30 |
| 199 | Turkey | 77 | 23 |
| 200 | Turkmenistan | 53 | 47 |
| 201 | Turks and Caicos Islands | 94 | 6 |
| 202 | Tuvalu | 65 | 35 |
| 203 | Uganda | 26 | 74 |
| 204 | Ukraine | 70 | 30 |
| 205 | United Arab Emirates | 87 | 13 |
| 206 | United Kingdom | 84 | 16 |
| 207 | United States | 83 | 17 |
| 208 | Uruguay | 96 | 4 |
| 209 | Uzbekistan | 50 | 50 |
| 210 | Vanuatu | 26 | 74 |
| 211 | Venezuela, RB | 88 | 12 |
| 212 | Vietnam | 38 | 62 |
| 213 | Virgin Islands (U.S.) | 96 | 4 |
| 214 | West Bank and Gaza | 77 | 23 |
| 215 | Yemen, Rep. | 39 | 61 |
| 216 | Zambia | 45 | 55 |
| 217 | Zimbabwe | 32 | 68 |
