# Supplementary material for: Socioeconomic disparities in mortality from indoor air pollution: A multi-country study
Source: PLoS One. 2025 Jan 16;20(1):e0317581. doi: 10.1371/journal.pone.0317581 (PMC11737656; doi:10.1371/journal.pone.0317581)
Supplement: S3 Table — (DOCX) [file pone.0317581.s003.docx]

**S3 Table.** Descriptive statistics of independent variables by country

| S. No | Country | People using safely managed sanitation services | Prevalence of Malnutrition | Total alcohol consumption per capita | Poverty headcount ratio | Population living in household with handwashing facility | Population living in houses with dung floors | Households with more than 7 persons sleeping per room | Household air pollution attributable deaths | Prevalence of current tobacco use  (% of adults) | Access to clean fuels and technologies for cooking  (% of population) |
| --- | --- | --- | --- | --- | --- | --- | --- | --- | --- | --- | --- |
| 1 | Afghanistan |  | 26 | 0.2 | 54.5 | 51.1 | 0.1 | 5.7 | 24,889 | 23.3 | 33 |
| 2 | Albania | 48 | 4 | 7.2 | 14.3 |  |  | 0.1 | 2028 | 22.4 | 81 |
| 3 | Algeria | 18 | 3 | 0.9 | 5.5 |  |  |  | 667 | 21.0 | 100 |
| 4 | American Samoa |  |  |  |  |  |  |  |  |  |  |
| 5 | Andorra | 100 |  | 11 |  |  |  |  | 13,761 | 31.8 | 100 |
| 6 | Angola |  | 17 | 6.9 | 32.3 |  | 0.1 | 3.7 | 0 |  | 50 |
| 7 | Antigua and Barbuda |  |  | 6.4 |  |  |  |  |  |  | 100 |
| 8 | Argentina | 51 | 4 | 9.7 | 42.9 |  |  |  | 848.0 | 24.5 | 100 |
| 9 | Armenia | 69 | 3 | 5.5 | 26.4 | 2.9 | 0.2 | 0.1 | 280 | 25.5 | 98 |
| 10 | Aruba |  |  |  |  |  |  |  |  |  |  |
| 11 | Australia | 74 | 3 | 10.5 |  |  |  |  | 0 | 13.6 | 100 |
| 12 | Austria | 100 | 3 | 12 | 13.3 |  |  |  | 0 | 26.4 | 100 |
| 13 | Azerbaijan | 21 | 3 | 4.4 | 6 |  |  | 0.8 | 1297 | 24.0 | 97 |
| 14 | The Bahamas |  |  | 4.8 |  |  |  |  | 0 | 10.6 | 100 |
| 15 | Bahrain | 91 |  | 1.1 |  |  |  |  | 0 | 14.9 | 100 |
| 16 | Bangladesh | 39 | 10 | 0 | 24.3 | 58.3 | 0 | 0.5 | 117,205 | 34.7 | 25 |
| 17 | Barbados |  | 4 | 9.7 |  |  |  |  | 0 | 8.5 | 100 |
| 18 | Belarus | 74 | 3 | 11.4 | 4.8 |  |  |  | 504.8 | 30.5 | 100 |
| 19 | Belgium | 89 | 3 | 11.1 | 14.8 |  |  |  | 0 | 23.4 | 100 |
| 20 | Belize |  | 6 | 6.2 |  |  |  |  | 58.41 | 8.5 | 82 |
| 21 | Benin |  | 8 | 2.8 | 38.5 | 47.9 | 1.2 | 1.8 | 9648 | 6.9 | 4 |
| 22 | Bermuda |  |  |  |  |  |  |  |  |  |  |
| 23 | Bhutan | 65 |  | 0.4 | 8.2 |  |  |  | 277.7 |  | 80 |
| 24 | Bolivia | 53 | 13 | 4.4 | 37.2 |  |  | 3.2 | 3159 | 12.7 | 86 |
| 25 | Bosnia and Herzegovina | 40 | 3 | 7.2 | 16.9 |  |  |  | 4933 | 35.0 | 45 |
| 26 | Botswana |  | 29 | 6.6 | 19.3 |  |  |  | 1291 | 19.4 | 65 |
| 27 | Brazil | 49 | 3 | 7.4 |  |  |  | 0.6 | 16,962 | 12.8 | 96 |
| 28 | British Virgin Islands |  |  |  |  |  |  |  |  |  |  |
| 29 | Brunei Darussalam |  | 3 | 0.5 |  |  |  |  | 0 | 16.2 | 100 |
| 30 | Bulgaria | 72 | 3 | 12.7 | 23.8 |  |  |  |  | 39.0 | 89 |
| 31 | Burkina Faso |  | 14 | 12 | 41.4 | 80.5 | 6.7 | 0.9 | 13,291 | 14.3 | 11 |
| 32 | Burundi |  |  | 7.2 | 64.9 | 93 | 0.9 | 0.3 | 7626 | 11.8 | 0 |
| 33 | Cabo Verde |  | 15 | 5.6 | 35 |  |  |  | 156.1 | 11.4 | 81 |
| 34 | Cambodia |  | 6 | 6.6 | 17.7 | 16.7 | 0 | 7.7 | 11,693 | 21.1 | 37 |
| 35 | Cameroon |  | 5 | 5.7 | 37.5 | 61.8 | 0.1 | 1 |  | 7.3 | 22 |
| 36 | Canada | 84 | 3 | 8.9 |  |  |  |  | 0 | 13.0 | 100 |
| 37 | Cayman Islands |  |  |  |  |  |  |  |  |  |  |
| 38 | Central African Republic | 14 | 48 | 2.4 | 62 |  |  | 1.6 | 5564 |  | 1 |
| 39 | Chad | 10 | 32 | 1.4 | 42.3 | 22.4 | 1.3 | 4 | 14,127 | 8.3 | 7 |
| 40 | Channel Islands | 82 |  |  |  |  |  |  |  |  |  |
| 41 | Chile | 79 | 3 | 9.1 | 10.8 |  |  |  | 0 | 29.2 | 100 |
| 42 | China | 70 | 3 | 7 | 0.6 |  |  |  | 727,845 | 25.6 | 79 |
| 43 | Colombia | 18 | 9 | 5.7 | 42.5 |  |  | 0.4 | 4935 | 8.5 | 93 |
| 44 | Comoros |  |  | 0.7 | 42.4 | 37.4 | 2.2 | 2.4 | 576.5 | 20.3 | 8 |
| 45 | Dem. Rep. Congo | 13 | 42 | 2 | 63.9 |  | 1 | 3.3 | 71,985 | 12.8 | 4 |
| 46 | Congo, Rep. |  | 38 | 9.3 | 40.9 | 12.2 |  | 1.4 | 2790 | 14.5 | 35 |
| 47 | Costa Rica | 30 | 3 | 4.9 | 30 |  |  |  | 223.1 | 8.8 | 96 |
| 48 | Cote d'Ivoire |  | 15 | 2.7 | 39.5 | 45.2 | 0.4 | 1.8 | 16,210 | 9.4 | 32 |
| 49 | Croatia | 68 | 3 | 9.2 | 18.3 |  |  |  | 0 | 36.9 | 100 |
| 50 | Cuba | 37 | 3 | 5.8 |  |  |  |  | 2812 | 17.9 | 94 |
| 51 | Curacao |  |  |  |  |  |  |  |  |  |  |
| 52 | Cyprus | 77 | 3 | 10.8 | 14.7 |  |  |  | 0 | 35.1 | 100 |
| 53 | Czech Republic | 85 | 3 | 14.4 | 10.1 |  |  |  | 0 | 30.7 | 100 |
| 54 | Denmark | 92 | 3 | 10.3 | 12.5 |  |  |  | 0 | 17.5 | 100 |
| 55 | Djibouti | 37 | 16 | 0.4 | 21.1 |  |  |  | 873.6 |  | 10 |
| 56 | Dominica |  | 6 | 11.2 |  |  |  |  |  |  | 89 |
| 57 | Dominican Republic |  | 8 | 6.7 | 21 |  |  | 0.4 | 1636 | 10.6 | 92 |
| 58 | Ecuador | 42 | 12 | 4.2 | 33 |  |  |  | 1073 | 11.3 | 94 |
| 59 | Egypt, Arab Rep. | 67 | 5 | 0.4 | 32.5 | 10.8 |  | 0.3 | 975.6 | 24.3 | 100 |
| 60 | El Salvador |  | 9 | 3.9 | 26.2 |  |  |  | 828.5 | 7.9 | 92 |
| 61 | Equatorial Guinea |  |  | 7.2 | 76.8 |  |  |  | 759.5 |  | 25 |
| 62 | Eritrea |  |  | 1.4 | 69 |  | 9.6 | 13.4 | 3766 | 7.5 | 9 |
| 63 | Estonia | 93 | 3 | 9.2 | 21.7 |  |  |  | 0 | 29.7 | 100 |
| 64 | Eswatini |  | 12 | 10 | 58.9 |  | 9.6 | 1.7 | 786.8 | 9.2 | 55 |
| 65 | Ethiopia | 7 | 16 | 2.4 | 23.5 | 52 | 9.9 | 11.5 | 63,027 | 5.1 | 8 |
| 66 | Faroe Islands |  |  |  |  |  |  |  |  |  |  |
| 67 | Fiji |  | 6 | 3.3 | 29.9 |  |  |  | 589 | 23.1 | 51 |
| 68 | Finland | 84 | 3 | 10.8 | 12.2 |  |  |  | 0 | 21.6 | 100 |
| 69 | France | 79 | 3 | 12.3 | 13.6 |  |  |  | 0 | 33.4 | 100 |
| 70 | French Polynesia |  | 4 |  |  |  |  |  |  |  |  |
| 71 | Gabon |  | 16 | 8.7 | 33.4 |  |  | 0.7 | 328.9 |  | 88 |
| 72 | Gambia, The | 29 | 14 | 3.5 | 48.6 | 84.6 | 0.3 | 0.7 | 1462 | 11.1 | 2 |
| 73 | Georgia | 34 | 9 | 8.3 | 21.3 |  |  |  | 2151 | 31.7 | 89 |
| 74 | Germany | 97 | 3 | 12.9 | 14.8 |  |  |  | 0 | 22.0 | 100 |
| 75 | Ghana | 13 | 6 | 2.8 | 23.4 | 35.6 | 0.1 | 1.6 | 18,881 | 3.5 | 22 |
| 76 | Gibraltar |  |  |  |  |  |  |  |  |  |  |
| 77 | Greece | 92 | 3 | 10.2 | 17.9 |  |  |  | 0 | 33.5 | 100 |
| 78 | Greenland | 92 |  |  |  |  |  |  |  |  |  |
| 79 | Grenada |  |  | 9.5 |  |  |  |  | 27.08 |  | 89 |
| 80 | Guam |  |  |  |  |  |  |  |  |  |  |
| 81 | Guatemala |  | 17 | 2.5 | 59.3 | 20 | 1.6 | 5.6 | 7460 | 10.9 | 50 |
| 82 | Guinea |  |  | 1.1 | 43.7 | 52.9 | 11.5 | 1.5 | 11,331 |  | 2 |
| 83 | Guinea-Bissau | 12 |  | 5.4 | 69.3 |  |  |  | 1425 | 9.0 | 1 |
| 84 | Guyana |  | 5 | 6.9 |  |  | 0.3 | 1.4 | 344.9 | 12.1 | 81 |
| 85 | Haiti |  | 47 | 2.7 | 58.5 | 63.2 | 0.8 | 1.9 | 13,323 | 7.7 | 5 |
| 86 | Honduras | 50 | 14 | 3.8 | 48 | 11.1 |  | 3 | 4740 |  | 48 |
| 87 | Hong Kong SAR, China | 86 | 3 |  |  |  |  |  |  |  |  |
| 88 | Hungary | 88 | 3 | 11.3 | 12.3 |  |  |  | 0 | 31.8 | 100 |
| 89 | Iceland | 84 | 3 | 9.1 | 8.8 |  |  |  | 0 | 12.0 | 100 |
| 90 | India | 46 | 15 | 5.5 | 21.9 | 37.9 | 0.7 | 3.2 | 811,262 | 27.2 | 68 |
| 91 | Indonesia |  | 7 | 0.6 | 9.4 | 6.4 | 0 | 0.4 | 92,898 | 37.6 | 85 |
| 92 | Iran, Islamic Rep. |  | 6 | 1 |  |  |  |  | 5890 | 13.6 | 96 |
| 93 | Iraq | 43 | 38 | 0.4 | 18.9 |  |  |  | 730 | 18.5 | 99 |
| 94 | Ireland | 83 | 3 | 12.9 | 13.1 |  |  |  | 0 | 20.8 | 100 |
| 95 | Isle of Man |  |  |  |  |  |  |  |  |  |  |
| 96 | Israel | 95 | 3 | 4.2 |  |  |  |  | 0 | 21.2 | 100 |
| 97 | Italy | 96 | 3 | 7.8 | 20.1 |  |  |  | 0 | 23.1 | 100 |
| 98 | Jamaica |  | 8 | 4.2 | 19.9 |  |  |  | 560.1 | 9.4 | 83 |
| 99 | Japan | 81 | 3 | 8 |  |  |  |  | 0 | 20.1 | 100 |
| 100 | Jordan | 82 | 10 | 0.7 | 15.7 |  |  | 1.5 | 50.91 | 34.8 | 100 |
| 101 | Kazakhstan |  | 3 | 4.8 | 4.3 |  |  | 0.1 | 3417 | 23.2 | 93 |
| 102 | Kenya |  | 25 | 2.8 | 36.1 | 24.8 | 8.1 | 1.8 | 22,283 | 11.1 | 20 |
| 103 | Kiribati | 27 | 4 | 0.5 | 21.8 |  |  |  | 156 | 40.6 | 10 |
| 104 | Korea, Dem. People's Rep. |  | 42 | 3.8 |  |  |  |  | 50,143 | 17.4 | 12 |
| 105 | Korea, Rep. | 100 | 3 | 9.7 |  |  |  |  | 0 | 20.8 | 100 |
| 106 | Kosovo |  |  |  | 17.6 |  |  |  |  |  |  |
| 107 | Kuwait | 100 | 3 | 0 |  |  |  |  | 0 | 17.9 | 100 |
| 108 | Kyrgyz Republic | 92 | 7 | 6.3 | 25.3 | 14.1 | 0.1 | 0.3 | 1862 | 25.4 | 77 |
| 109 | Lao PDR | 61 | 5 | 10.7 | 18.3 |  |  |  | 6054 | 31.8 | 9 |
| 110 | Latvia | 83 | 3 | 12.8 | 22.9 |  |  |  | 0 | 37.0 | 100 |
| 111 | Lebanon | 16 | 9 | 1.7 | 27.4 |  |  |  |  | 38.2 |  |
| 112 | Lesotho | 48 | 24 | 4.6 | 49.7 | 43 |  | 0.7 | 3229 | 24.3 | 40 |
| 113 | Liberia |  | 39 | 6.1 | 50.9 | 23.3 | 1.3 | 1.4 | 2940 | 8.2 | 0 |
| 114 | Libya | 22 |  | 0 |  |  |  |  |  |  |  |
| 115 | Liechtenstein | 99 |  |  |  |  |  |  |  |  |  |
| 116 | Lithuania | 94 | 3 | 13.2 | 20.6 |  |  |  | 0 | 32.0 | 100 |
| 117 | Luxembourg | 97 | 3 | 12.9 | 17.5 |  |  |  | 0 | 21.1 | 100 |
| 118 | Macao SAR, China | 67 | 4 |  |  |  |  |  |  |  |  |
| 119 | Madagascar | 10 | 43 | 2 | 70.7 |  | 1 | 6.2 | 21,345 | 27.8 | 1 |
| 120 | Malawi | 24 | 17 | 3.6 | 51.5 | 79.2 | 6.4 | 2 | 8822 | 10.8 | 1 |
| 121 | Malaysia | 77 | 3 | 0.8 | 8.4 |  |  |  | 4476 | 22.5 | 96 |
| 122 | Maldives |  |  | 2.2 | 8.2 | 1.8 |  | 0.9 | 8.3 | 25.2 | 99 |
| 123 | Mali | 20 | 10 | 1.3 | 41.9 | 60.9 | 6.5 | 1.2 | 11,859 | 8.3 | 1 |
| 124 | Malta | 92 | 3 | 8 | 17.1 |  |  |  | 0 | 24.0 | 100 |
| 125 | Marshall Islands |  |  |  |  |  |  |  |  | 28.5 | 64 |
| 126 | Mauritania |  | 9 | 0 | 31 |  |  | 17.5 | 1896 | 10.7 | 43 |
| 127 | Mauritius |  | 6 | 4.3 | 10.3 |  |  |  | 136.2 | 20.2 | 97 |
| 128 | Mexico | 57 | 7 | 5 | 43.9 |  |  |  | 21,639 | 13.1 | 85 |
| 129 | Micronesia, Fed. Sts. |  |  | 2.5 | 41.2 |  |  |  | 149.8 |  | 13 |
| 130 | Moldova |  |  | 11.4 | 26.8 |  |  |  | 731.8 | 29.0 | 96 |
| 131 | Monaco | 100 |  |  |  |  |  |  |  |  | 100 |
| 132 | Mongolia | 56 | 4 | 8.2 | 28.4 |  |  |  | 2420 | 29.4 | 52 |
| 133 | Montenegro | 45 | 3 | 11.5 | 24.5 |  |  |  | 771.4 | 31.4 | 62 |
| 134 | Morocco | 39 | 4 | 0.7 | 4.8 |  | 0.4 |  | 2878 | 14.5 | 98 |
| 135 | Mozambique |  | 31 | 2.3 | 46.1 | 33.1 | 4.2 | 1.4 | 26,452 | 14.3 | 5 |
| 136 | Myanmar | 61 | 8 | 5.1 | 24.8 | 15.3 | 0.1 | 3.1 | 54,038 | 44.1 | 31 |
| 137 | Namibia |  | 20 | 5.4 | 17.4 | 45.4 | 1.9 | 1.2 | 1434 | 15.1 | 47 |
| 138 | Nauru |  |  | 3.7 |  |  |  |  |  | 48.5 | 100 |
| 139 | Nepal | 49 | 5 | 2.9 | 25.2 | 53.9 | 7.3 | 1 | 25,069 | 30.4 | 35 |
| 140 | Netherlands | 97 | 3 | 9.6 | 13.6 |  |  |  | 0 | 22.2 | 100 |
| 141 | New Caledonia |  | 7 |  |  |  |  |  |  |  |  |
| 142 | New Zealand | 82 | 3 | 10.6 |  |  |  |  | 0 | 13.7 | 100 |
| 143 | Nicaragua |  | 19 | 5.2 | 24.9 |  |  | 13.3 | 2624 |  | 56 |
| 144 | Niger | 16 |  | 0.7 | 40.8 |  | 0 | 4.9 | 16,439 | 7.4 | 2 |
| 145 | Nigeria | 31 | 15 | 10.8 | 40.1 | 57.1 | 0.3 | 1.7 | 132,494 | 3.7 | 15 |
| 146 | North Macedonia | 12 | 3 | 6.2 | 21.6 |  |  |  | 1370 |  | 78 |
| 147 | Northern Mariana Islands |  |  |  |  |  |  |  |  |  |  |
| 148 | Norway | 65 | 3 | 7.4 | 12.7 |  |  |  | 0 | 16.2 | 100 |
| 149 | Oman |  | 8 | 0.8 |  |  |  |  | 0 | 8.0 | 100 |
| 150 | Pakistan |  | 13 | 0.3 | 21.9 | 31.7 | 2.7 | 10 | 152,925 | 20.2 | 49 |
| 151 | Palau |  |  |  | 24.9 |  |  |  |  | 17.6 | 100 |
| 152 | Panama |  | 8 | 8 | 22.1 |  |  |  | 546.3 | 5.0 | 88 |
| 153 | Papua New Guinea |  | 25 | 1.4 | 39.9 | 33.6 |  | 3.4 | 7295 | 39.3 | 9 |
| 154 | Paraguay | 60 | 9 | 7.6 | 26.9 |  |  |  | 2035 | 11.5 | 69 |
| 155 | Peru | 53 | 9 | 6.4 | 20.2 |  |  | 1 | 5345 | 8.1 | 85 |
| 156 | Philippines | 61 | 9 | 6.9 | 16.7 | 10.7 |  | 2.4 | 94,441 | 22.9 | 48 |
| 157 | Poland | 91 | 3 | 11.7 | 15.4 |  |  |  | 0 | 24.0 | 100 |
| 158 | Portugal | 85 | 3 | 12 | 17.2 |  |  |  | 0 | 25.4 | 100 |
| 159 | Puerto Rico | 33 |  |  |  |  |  |  |  |  |  |
| 160 | Qatar | 97 |  | 1.6 |  |  |  |  | 0 | 11.8 | 100 |
| 161 | Romania | 83 | 3 | 11.7 | 23.8 |  |  |  | 11,316 | 28.0 | 88 |
| 162 | Russian Federation | 61 | 3 | 11.2 | 12.1 |  |  |  | 85,329 | 26.8 | 86 |
| 163 | Rwanda |  | 35 | 8.9 | 38.2 | 59.3 | 0.2 | 0.2 | 6932 | 13.7 | 2 |
| 164 | Samoa | 48 | 5 | 2.7 | 20.3 |  |  |  | 153.9 | 25.3 | 37 |
| 165 | San Marino | 70 |  |  |  |  |  |  |  |  | 100 |
| 166 | Sao Tome and Principe | 35 | 12 | 5.9 | 66.7 |  | 0.8 | 1.2 |  | 5.7 | 3 |
| 167 | Saudi Arabia | 59 | 4 | 0.2 |  |  |  |  | 0 | 14.3 | 100 |
| 168 | Senegal | 24 | 8 | 0.8 | 46.7 | 26.3 | 1.2 | 1.3 | 7635 | 6.9 | 24 |
| 169 | Serbia | 18 | 4 | 8.8 | 23.2 |  |  |  | 5845 | 39.8 | 80 |
| 170 | Seychelles |  |  | 20.5 | 25.3 |  |  |  | 0 | 20.2 | 100 |
| 171 | Sierra Leone | 14 | 26 | 5.7 | 56.8 | 25.5 | 0.8 | 0.7 | 7334 | 13.5 | 1 |
| 172 | Singapore | 100 |  | 2 |  |  |  |  | 0 | 16.5 | 100 |
| 173 | Sint Maarten (Dutch part) |  |  |  |  |  |  |  |  |  |  |
| 174 | Slovak Republic | 82 | 4 | 11.1 | 11.9 |  |  |  | 0 | 31.5 | 100 |
| 175 | Slovenia | 72 | 3 | 11.9 | 12 |  |  |  |  | 22.0 | 100 |
| 176 | Solomon Islands |  | 17 | 1.8 | 12.7 |  |  |  | 829.7 | 36.5 | 9 |
| 177 | Somalia | 32 | 60 | 0 |  |  |  |  | 16,061 |  | 3 |
| 178 | South Africa |  | 7 | 9.5 | 55.5 | 46.5 | 3.1 | 0.6 | 14,390 | 20.3 | 87 |
| 179 | South Sudan |  |  |  | 76.4 |  |  |  | 7072 |  | 0 |
| 180 | Spain | 96 | 3 | 12.7 | 20.7 |  |  |  | 0 | 27.7 | 100 |
| 181 | Sri Lanka |  | 7 | 4.1 | 4.1 |  |  |  | 17,369 | 22.0 | 32 |
| 182 | St. Kitts and Nevis |  |  | 8.9 |  |  |  |  |  |  | 100 |
| 183 | St. Lucia |  |  | 10.6 | 25 |  |  |  | 20.78 |  | 94 |
| 184 | St. Martin (French part) |  |  |  |  |  |  |  |  |  |  |
| 185 | St. Vincent and the Grenadines |  | 6 | 9.1 |  |  |  |  | 15.07 |  | 94 |
| 186 | Sudan |  | 12 | 0.5 | 46.5 |  |  |  | 19,185 |  | 55 |
| 187 | Suriname | 25 | 9 | 5.3 |  |  |  |  | 72.19 |  | 95 |
| 188 | Sweden | 95 | 3 | 8.9 | 17.1 |  |  |  | 0 | 24.0 | 100 |
| 189 | Switzerland | 100 | 3 | 11.5 | 16 |  |  |  | 0 | 25.5 | 100 |
| 190 | Syrian Arab Republic |  |  | 0.2 | 35.2 |  |  |  | 1509 |  | 97 |
| 191 | Tajikistan |  |  | 3.3 | 26.3 | 23.1 |  | 0.5 | 2579 |  | 82 |
| 192 | Tanzania | 26 | 25 | 11.3 | 26.4 | 38.1 | 3 | 0.5 | 25,323 | 8.7 | 5 |
| 193 | Thailand | 26 | 8 | 8.3 | 6.2 |  |  |  | 22,836 | 22.1 | 84 |
| 194 | Timor-Leste |  | 23 | 2.2 | 41.8 | 67.1 | 0.6 | 1.4 | 1098 | 39.2 | 14 |
| 195 | Togo | 9 | 20 | 2.5 | 55.1 |  |  | 1.1 | 6087 | 6.8 | 10 |
| 196 | Tonga | 34 |  | 0.8 | 22.5 |  |  |  | 24.34 | 31.0 | 84 |
| 197 | Trinidad and Tobago |  | 7 | 6.7 |  |  |  |  | 0 |  | 100 |
| 198 | Tunisia | 81 | 3 | 2.1 | 15.2 |  |  |  | 181.2 | 24.6 | 100 |
| 199 | Turkey | 78 | 3 | 2 | 15 |  |  | 0.5 | 6679 | 30.7 | 95 |
| 200 | Turkmenistan |  | 4 | 4.9 |  |  |  |  | 65.4 | 5.5 | 100 |
| 201 | Turks and Caicos Islands |  |  |  |  |  |  |  |  |  |  |
| 202 | Tuvalu | 6 |  | 1.5 | 26.3 |  |  |  |  | 35.6 | 71 |
| 203 | Uganda |  |  | 15.1 | 20.3 | 41.4 | 19.8 | 2.8 | 18,649 | 8.4 | 1 |
| 204 | Ukraine | 72 | 3 | 8.3 | 1.1 |  | 0.1 | 0 | 13,781 | 25.8 | 95 |
| 205 | United Arab Emirates | 99 | 4 | 3.9 |  |  |  |  | 0 |  | 100 |
| 206 | United Kingdom | 98 | 3 | 11.4 | 18.6 |  |  |  | 0 | 15.4 | 100 |
| 207 | United States | 98 | 3 | 9.9 |  |  |  |  | 0 | 23.0 | 100 |
| 208 | Uruguay |  | 3 | 6.9 | 11.6 |  |  |  | 0 | 21.5 | 100 |
| 209 | Uzbekistan |  | 3 | 2.6 | 14.1 |  | 0.5 | 0.7 | 9398 | 17.6 | 84 |
| 210 | Vanuatu |  | 9 | 2.3 | 15.9 |  |  |  | 358 | 17.8 | 8 |
| 211 | Venezuela, RB | 23 | 27 | 4.1 | 33.1 |  |  |  | 1762 |  | 97 |
| 212 | Vietnam |  | 7 | 8.7 | 6.7 |  |  | 1.5 | 61,510 | 24.8 | 65 |
| 213 | Virgin Islands (U.S.) |  |  |  |  |  |  |  |  |  |  |
| 214 | West Bank and Gaza | 67 |  |  | 29.2 |  |  |  |  |  |  |
| 215 | Yemen, Rep. | 19 | 45 | 0.1 | 48.6 | 27.1 | 9.8 | 9 | 12,642 | 20.3 | 62 |
| 216 | Zambia |  |  | 6.5 | 54.4 | 39.4 | 4 | 2.2 | 9498 | 14.4 | 10 |
| 217 | Zimbabwe | 26 |  | 4.7 | 38.3 | 62 | 11.7 | 0.6 | 9738 | 11.7 | 30 |
